# DISCOVERY: a study examining the prevalence of transthyretin mutations in subjects suspected of having cardiac amyloidosis

**DOI:** 10.1186/1750-1172-10-S1-O8

**Published:** 2015-11-02

**Authors:** Olakunle Akinboboye, Karthik Ananthasubramaniam, Amir Malik, Alberta Warner, Verena Karsten, Thibaud Damy, Herman A Taylor, Mathew S Maurer

**Affiliations:** 1 Queens Heart Institute, Medicine, 11422, Rosedale, USA; 2 Henry Ford Hospital, Heart and Vascular Institute, 48202, Detroit, USA; 3 Heart Center of North Texas, Cardiology, 76104, Fort Worth, USA; 4 Los Angeles Veterans Affairs Health Care System, Echo lab and HF service, 90073, Los Angeles, USA; 5 Alnylam Pharmaceuticals, Clinical Development, 02142, Cambridge, USA; 6 APHP, Cardiology and Amyloidosis Mondor Network, 94000, Creteil, France; 7 Morehouse School of Medicine, Cardiovascular Research Institute, 30310, Atlanta, USA; 8 Columbia University Medical Center, Medicine, 10032, New Yok, USA

## Background

Cardiac amyloidosis (CA) is caused by extracellular myocardial deposition of either immunoglobulin light-chain (AL) or transthyretin (ATTR) fibrils. Two forms of ATTR CA cause life-threatening cardiomyopathy: an inherited form arising from misfolding of mutated ATTR (familial amyloid cardiomyopathy [FAC]) and a sporadic form caused by wild-type ATTR (senile systemic amyloidosis [SSA]). More than half of over 100 reported ATTR mutations are associated with FAC. The most common mutation in the US is Val122Ile found in 3-4% of African Americans (AA). FAC can be difficult to recognize clinically and is likely under diagnosed. The DISCOVERY study aims to determine the prevalence of TTR mutations and FAC diagnosis in a cohort of patients (pts) with clinical features suggestive of CA.

## Methods

This is a prospective, multi-center study in adults with two or more of the following eligibility criteria: heart failure signs and symptoms, intraventricular septal thickness (IVS) of >12 mm, LV diastolic dysfunction, low voltage ECG, or history of carpal tunnel disease (CTD). DNA from blood samples is used for sequencing of the TTR gene coding regions by a central lab. Assessments in pts with TTR mutations include cardiac biomarkers, echocardiogram and optional abdominal fat pat aspiration and 6-minute walk test. Descriptive statistics will be utilized.

## Results

As of May 2015, 146 pts have been enrolled. At baseline, the mean (Std) age of pts is 64 (13) yrs, 61% are men and 66% are AA. A total of 14 (10%) pts had a Val122Ile mutation and 1 pt had a novel mutation Arg103His. The Gly6Ser polymorphism was found in 8 (5%) pts. The Val122Ile cohort consisted of 40% males with a mean (StD) age of 66 (16). Heart failure signs and symptoms and IVS > 12 mm was reported in 71% and 79% of Val122IIle pts respectively. The majority of pts had NYHA class II (56%) and III (22%) heart failure, 21% had low voltage ECGs and 14% had CTD.

## Conclusions

These preliminary data suggest that approximately 10% of pts with clinical and/or radiologic findings suggestive of cardiac amyloidosis have a pathogenic TTR mutation which could potentially lead to a diagnosis of FAC. Additional data on clinical features and tissue diagnosis of FAC in these pts will be presented.

